# Variation in processes of care for total hip arthroplasty across high-income countries

**DOI:** 10.1093/haschl/qxae043

**Published:** 2024-04-24

**Authors:** Laura Skopec, Robert A Berenson, Benedikt Simon, Irene Papanicolas

**Affiliations:** Health Policy Center, Urban Institute, Washington, DC 20037, United States; Health Policy Center, Urban Institute, Washington, DC 20037, United States; Department for Integrated and Digital Care, Asklepios Kliniken GmbH & Co KGaA, 22307 Hamburg, Germany; Department of Health Services, Policy & Practice, Brown University School of Public Health, Providence, RI 02903, United States

**Keywords:** international comparisons, hip replacement, total hip arthroplasty, processes of care

## Abstract

Total hip arthroplasty (THA) is among the most commonly performed elective surgeries in high-income countries, and wait times for THA have frequently been cited by US commentators as evidence that countries with universal insurance programs or national health systems “ration” care. This novel qualitative study explores processes of care for hip replacement in the United States and 6 high-income countries with a focus on eligibility, wait times, decision-making, postoperative care, and payment policies. We found no evidence of rationing or government interference in decision-making across high-income countries. Compared with the 6 other high-income countries in our study, the United States has developed efficient care processes that often allow for a same-day discharge. In contrast, THA patients in Germany stay in the hospital 7–9 days and receive 2–3 weeks of inpatient rehabilitation. However, the payment per THA in the United States remains far above other countries, despite far fewer inpatient days.

## Introduction

Total hip arthroplasty (THA), is one of the most common, high-cost elective procedures across high-income countries.^1^ In the United States, THA was the fourth most common operating room procedure performed during inpatient stays in 2018,^2^ and hip and knee replacements are the most common surgical procedure covered by Medicare.^3^ As populations across countries age, spending on THAs is expected to grow significantly.^4-6^

United States’ politicians and commentators frequently cite difficulties getting elective care and procedure wait times in other countries as evidence that universal insurance programs or national health systems “ration” care.^7-9^ While some existing research compares the use and outcomes of THA across countries, few studies focus on comparing processes of care for THA in the United States with other high-income countries.^1,10-14^

A 2014 article compared elective-procedure waiting times across selected high-income countries in Europe, including THA.^15^ This work formed the basis of the current THA waiting times reported annually by the Organization for Economic Cooperation and Development (OECD). A 2019 study compared patient characteristics, surgical techniques, and implants across Australia, Sweden, and a US sample from Kaiser Permanente and found differences in the types of implants used across the 3 countries, but the study did not focus on eligibility, waiting times, or other processes of care.^11^ Other comparative work, between England and Scotland, showed that changes in reimbursement rates can influence implant choices even across countries where clinical guidelines are the same.^16^ Finally, a recent paper compared processes of care for THA between Germany and a US sample from Kaiser Permanente and found significant variation in the length of THA hospitalization and approaches to post-acute care.^17^ However, this study was limited to just 2 countries.

The current study expands on prior research by using a vignette approach to document a variety of care processes across 7 high-income countries with different organizational structures to better understand what might contribute to differences in THA uptake and outcomes.

## Data and methods

This study used qualitative methods, supplemented with secondary data sources and literature, to explore variation in care processes for uncomplicated THA across 7 countries: Canada, England, France, Germany, New Zealand, Norway, and the United States. These countries all have high health care spending and use a mix of funding models.^10^ They also have differing THA rates, eligibility rules, waiting times, and post-acute care approaches.^1,10-14,17,18^ The 7 selected countries were drawn from those included in the Commonwealth Fund's International Program,^19^ and the lead contacts in each were a convenience sample of the authors’ and funders’ contacts.

We fielded an 8-page questionnaire to 2–4 experts in each country of interest from March 2023 (Appendix S1; to access the Appendix, click on the Details tab of the article online) identified by our lead contact in each country. Experts included health care professionals (eg, orthopedic surgeons, physiotherapists) and national researchers in joint replacement, and included regional mix of respondents where possible (Appendix S2). The questionnaire focused on the care process of a standardized patient: a 68-year-old male with osteoarthritis and no complicating chronic conditions covered by the dominant public insurance program in the country or region (Medicare in Ontario and Nova Scotia, Canada; National Health Insurance Fund in France; Public Health Insurance [Gesetzliche Krankenversicherung] in Germany; public health care in New Zealand; Folketrygd in Norway; National Health Service in England; and Medicare in the United States).

The questionnaire focused on topics including eligibility, waiting lists, surgical and device approaches, length of hospital stay (LOS), post-acute care, outcomes tracking, and the payment system. We also asked respondents to describe variations in care for patients with comorbidities, private insurance, or access to private hospitals.

We supplemented our questionnaires with data on THA rates, waiting lists, and outcomes from the OECD Health database and published national statistics from the respective countries. We identified THA payment rates where available, but comparable data were only available for a limited number of countries.

### Limitations

This study has several limitations. Our expert questionnaires and secondary data gathering were limited to a convenience sample of 7 countries. This study therefore does not represent a comprehensive picture of how processes of care for THA may vary, although the countries do represent a mix of system types. We also gathered questionnaires from only 2–4 experts per country, which may not adequately describe within-country regional or health system variation. For example, in the United States, our experts were primarily drawn from academic health centers, which may have different approaches than rural or safety-net hospitals. While we asked experts to support their responses with literature or links to public data sources, in some cases it was difficult to reconcile the variations in responses among experts in a country, and those questionnaire responses had to be dropped from our analysis.

We also did not collect new data on costs or outcomes, instead relying on literature review and secondary sources. Unfortunately, publicly reported data on costs and outcomes are not always comparable across countries. On wait times, we asked experts to identify the amount of time between surgical referral and surgery, which does not account for differences in wait times for primary care visits or referrals to specialists. We also did not ask experts to describe processes for presurgical management of osteoarthritis, so our results cannot speak to how primary care processes may affect differences in THA rates and surgical choices.

This study has limitations common to qualitative studies, including that the responses of the experts may not be representative, that some responses may reflect expert opinions or approximations rather than national data, and that our findings do not imply any causal relationship between hip-replacement processes of care and variations in outcomes across countries. Where possible, questionnaire responses were verified with public data sources or published literature to improve accuracy.

## Results

### THA rates

Recent OECD data on the number of THAs performed per 100 000 population are available for all countries except the United States. In 2019, rates varied from 314.9 THAs per 100 000 population in Germany to 159.6 per 100 000 population in New Zealand (Table 1). Data from the Healthcare Cost and Utilization Project show that the United States performed 232.1 THAs per 100 000 in 2019,^20^ although this includes only hospital inpatient procedures.

**Table 1.** Volume of hip replacements per 100 000 population in selected high-income countries, 2019–2021.

	Total hip replacements per 100 000 population			Inpatient hip replacements per 100 000 population: 2019
	2019	2020	2021	
Canada	168.1	144.6	152.7	164.6
France	251.5	220.8	245.8	246.7
Germany	314.9	294.1	300.8	314.8
New Zealand	159.6	146.9	N/A	158.8
Norway	267.6	239.6	255.3	266.7
United Kingdom	182.4	97.6	180.3	181.5
United States	N/A	N/A	N/A	232.1^a^

Abbreviations: N/A, not available; OECD, Organization for Economic Cooperation and Development.

Sources: United States: McDermott and Liang.^2^ Other countries: OECD.^21^ Data are not adjusted for differences in age or health status across countries.

^a^US data are from a different source and may not be fully comparable to OECD data on inpatient hip replacements.

In all countries except Canada and the Untied States, nearly all THAs were performed in inpatient settings. Since 2019, the share of THAs performed as outpatient procedures in the United States and Canada has increased markedly, perhaps partially due to the pandemic.^22,23^

### Coverage

Across all study countries, a 68-year-old male with uncomplicated osteoarthritis is typically covered by public insurance. However, countries vary in the role that private hospitals and private insurance play in THA. Some countries allow the purchase of private insurance that may cover THAs or help defray cost-sharing for private hospital procedures. In France, for example, public health insurance covers THAs performed in public and private hospitals equally, but complementary private insurance may cover private rooms or cost-sharing. In New Zealand, a significant minority of THAs are covered by private insurance or in private hospitals. In England, patients can pay fully out-of-pocket for privately provided THAs. In the United States, many THAs are covered by Medicare Advantage, which is an optional program that allows private health insurance plans to cover Medicare benefits for enrollees. Medicare Advantage plans generally pay facility and physician fees close to those in traditional Medicare.^24^

### Referral for surgery

Our results suggest that referral processes were similar across the study countries (Table 2). For all but 1 country, primary care physicians generally refer patients to surgeons or surgical practices for THA. In Ontario and Nova Scotia, Canada, primary care physicians instead refer patients to central provincial (or regional) intake, which confirms eligibility and sets waitlist priority. Experts in the United States, Germany, and France indicated that patients can also self-refer to a surgeon or surgical practice. In England and New Zealand, patients denied surgery in the public health system or who do not wish to wait for surgery can self-refer to a private hospital, but they may be responsible for paying all costs out-of-pocket.

**Table 2.** Referral processes and selection of prosthesis and surgical approach for THA in select high-income countries.

	Typical referral process for THA	Who decides procedure type?	Who selects prosthesis type?
Canada	Primary care physician refers to central intake	Surgeon	Surgeon
France	Primary care physician refers to surgeon or patient self refers	Surgeon	Surgeon or joint decision with patient
Germany	Primary care physician refers or patient self-refers	Surgeon	Surgeon, and hospitals may have contracts with manufacturers
New Zealand	Primary care physician refers to surgeon or practice	Surgeon	Surgeon or joint decision with patient
Norway	Primary care physician refers to surgeon or practice	Surgeon, hospital guidelines	Surgeon, hospital guidelines, national/regional guidelines and purchasing programs
United Kingdom	Primary care physician refers to surgeon, practice, or hospital	Surgeon	Surgeon with some payer guidelines/limits
United States	Primary care physician refers or patient self-refers	Surgeon	Surgeon, with guidelines at some hospitals

Abbreviation: THA, total hip arthroplasty.

Source: Expert reports of typical processes for a 68-year-old male with osteoarthritis and no complicating chronic conditions.

### THA eligibility

Experts in England, France, Germany, Norway, and the United States indicated that THA eligibility is based primarily on a physician's recommendation or certification. In many countries, including the United States, guidelines are in place to help determine surgical candidates,^25,26^ although our experts generally did not mention or emphasize these guidelines in their responses about eligibility. In Ontario and Nova Scotia, Canada, the central intake program confirms eligibility. In New Zealand, there is a threshold scoring system based on symptom severity for the public waitlist, but private surgery is available as an alternative.

In the United States, respondents noted that insurance companies, like Medicare Advantage, generally want to see 12 weeks of conservative treatment and documentation of arthritis severity in the patient's chart before approving surgery. This is consistent with Medicare's coverage guidelines.^27^

In Canada, England, France, Germany, and Norway, patients who are determined to be eligible for THA are never or rarely denied coverage for the procedure. In the United States, coverage denials are more common and may be related to inadequate documentation^28^ or Medicare Advantage coverage issues like prior authorization requirements.^29^

### Wait times

Patients in all 7 study countries face wait times for THA surgery, but these wait times vary by region, hospital, and coverage type. Only Canada, England, New Zealand, and Norway publicly report wait times for THA (Table 3). Across these countries, reported wait times between specialist referral and surgery averaged 3–6 months in 2018, but wait times have been increasing since the COVID-19 pandemic.

**Table 3.** Wait times between specialist referral and surgery for THA in select high-income countries.

	Median wait time in 2018, OECD	Median wait time in 2021, OECD	Share of patients waiting more than 3 months in 2018, OECD	Share of patients waiting more than 3 months in 2021, OECD	Average wait time in 2023, expert reports^a^
Canada	105 days	134 days			3–6 months. One expert in a rural area reported up to 1.5 years.
France	France does not report these data to the OECD				Experts said wait times are not tracked.
Germany	Germany does not report these data to the OECD.				One expert reported 2–4 weeks; 1 expert reported 2 weeks–6 months
New Zealand	81 days	112 days	42.5%	62.3%	6–12 months for public hospitals; 1–6 weeks for private
Norway^b^	123 days	151 days	68.0%	79.3%	6 months
United Kingdom	92 days	N/A	51.3%		Two experts reported 12 months; 1 expert reported 3 months
United States	The United States does not report these data to the OECD.				2–6 months

Abbreviations: N/A, not available; OECD, Organization for Economic Cooperation and Development; THA, total hip arthroplasty.

Source: expert reports and OECD.^21^

^a^Experts across all countries noted that wait times vary by hospital, region, and sometimes by surgeon.

^b^Norway calculates wait times from general practitioner (GP) referral to treatment in OECD data reporting, so estimates are overstated relative to other countries, which report wait times between specialist referral and surgery.

Our expert respondents also indicated that wait times are often in the range of 3–6 months in Canada, Norway, and the United States. (In the United States, our experts were concentrated in academic medical centers, which may have longer wait times for THA than other hospitals.) In England, experts reported wait times of up to 1 year for publicly funded surgery, although 1 expert reported a 3-month average wait time. In New Zealand, experts reported wait times of between 4 and 18 months, with regional variability. Across countries, experts noted that wait times vary substantially by region, hospital, and surgeon, and that the COVID-19 pandemic increased wait times. This may account for differences between expert reports and the OECD reports, which are based on pre-pandemic data.

In England and New Zealand, experts also noted that wait times are significantly shorter for private hospitals than for public ones. In Germany, no wait times are reported to OECD, and our experts reported wait times of as little as 2 weeks to a few months. Both are consistent with excess hospital capacity in Germany that would lead to little to no wait time,^30^ particularly for those with private insurance.^31^

### Selection of prosthesis and surgical approach

According to our experts, surgeons are primarily responsible for selecting the THA surgical approach (Table 2). In Norway, hospital guidelines for THA also play a role in determining the options for surgical approach. Experts also indicated that hospitals, payers, and governments were more involved in the selection of prosthesis, often because of bulk purchasing programs to lower costs (Table 2). US demonstration programs have shown savings from collaborative, physician-led processes to select cost-effective medical devices for standard usage.^32^

### Hospital LOS

Across 6 of the 7 countries, experts indicated that hospital stays are relatively short, generally less than 3 days (Table 4). In the United States and Canada, experts said that patients are often discharged the same day with no overnight stay. Lengths of stay for THA have been falling in many countries over the past decade,^33^ including the United States^34,35^ and Canada.^36,37^ In contrast, hospital stays for THA in Germany remain long, averaging about 7–9 days.^17,38^

**Table 4.** Typical length of hospital stay and hospital and physician reimbursement for uncomplicated THA in selected countries.

	Typical length of stay	THA reimbursement, including facility and physician fee (in USD)
Canada	0–1 days	$7709 (2021/2022)
France	1–5 days	N/A
Germany	8–10 days	$7560 (2023)
New Zealand	2–3 days	N/A
Norway	2–3 days	N/A
United Kingdom	0–3 days	N/A
United States	0–1 days	$14 348 (2023)

Abbreviations: N/A, not available; THA, total hip arthroplasty; USD, US dollars.

Sources: Reimbursement data from the following: Canada, Canadian Institute for Health Information^23^; Germany, Waldemar Link GmbH^38^; United States, Centers for Medicare & Medicaid Services (CMS).^39^ Length of stay data from expert reports of typical length of stay for a 68-year-old male with osteoarthritis and no complicating chronic conditions after successful THA. Reimbursement in US dollars. Canadian dollars and Euros were converted to US dollars using the conversion rates 0.74 and 1.09, respectively.

### Types of post-acute care

In Canada, England, France, New Zealand, Norway, and the United States, our experts indicated that the vast majority of patients are discharged home after THA. In Germany, over 90% of patients are discharged to an inpatient rehabilitation facility, where they stay approximately 2–3 weeks.^17^ Germany residents are entitled by law to inpatient rehabilitation after hospitalization for THA.^40^

Across 6 of the 7 countries, experts indicated that a 68-year-old male with osteoarthritis and no complicating conditions would receive physical therapy at home or in an outpatient setting after a successful THA. The number of physical therapy visits provided can vary widely based on patient needs. Additionally, the timing of physical therapy is highly variable, particularly in rural areas. Experts in New Zealand noted that physical therapy is not typically provided after hospital discharge. Instead, patients in New Zealand receive guidance in self-directed exercises to complete during recovery.

### Payment systems

The predominant payment model varied across countries, possibly altering incentives to provide and carry out THA. Most countries have a combination of salaried physicians with diagnosis-related group (DRG) payments under global budgets or caps (England, France, Germany, and Norway).^41^ A similar approach applies in New Zealand, where public hospitals are under global budgets with salaried physicians. Private hospitals may pay differently across systems, such as in France, offering a fee-for-service payment for each THA.

Our experts in Ontario and Nova Scotia, Canada, noted that hospitals receive a bundled payment for an episode of care for THA, and that target THA volumes are set by the government. If hospitals go over their target volumes, their episode payments are adjusted downward. Physicians in Canada are paid fee-for-service for THA, which would tend to increase incentives to do more surgeries.

In the United States, hospitals receive a DRG payment from Medicare for each THA performed, and the physicians also receive fee-for-service payments for each procedure, although some alternative payment models are underway.

Overall, DRG payments for THA would tend to increase hospital willingness to perform greater volumes of surgeries to increase reimbursements, but global budgets or caps would reduce that incentive. Furthermore, salaried physicians likely have weaker financial incentives to increase THA volume as compared with those paid on a fee-for-service basis. However, hospitals may still pressure physicians to increase volume in these systems through targets or other means.

Our experts reported that many countries are testing new payment approaches for THA or creating incentives to improve value in THA. In England, hospitals can receive quality bonuses for THA under the Best Practice Tariff.^42^ In addition, our French and German experts reported that there are experiments with bundled payments, quality contracts, and other approaches to improve quality underway. In the United States, the Centers for Medicare and Medicaid Innovation (CMMI) are testing models to improve value in THA. The current model, called Comprehensive Care for Joint Replacement, has been underway since 2016. This model holds participating hospitals accountable for improving quality and reducing costs for hip and knee replacements. As of 2019, this program had produced an estimated $72 million in cumulative savings through small reductions in per-case costs, but the COVID-19 pandemic and related relaxations in penalties resulted in excess Medicare costs of $23.4 million in 2020.^43^

The average Medicare payment in the United States for an uncomplicated THA performed in a hospital outpatient department in 2023 was $14 348, including a $1300 physician fee and a $13 048 facility fee (Table 4).^39^ In contrast, the hospital DRG payment for an uncomplicated THA in Germany in 2023, including an average 7.2-day hospital stay post-surgery, was €7293.40 or approximately $7560 US dollars (USD; conversion rate of 1.09). In Ontario, Canada, the estimated inpatient facility cost for hip replacement in 2021–2022 was $7706 Canadian,^23^ or about $5702 USD (conversion rate of 0.74), not including physician fees of approximately $2007 Canadian ($1485 USD).

While the years and contents of these payments differ, the United States is clearly an outlier in payment for THA. US Medicare payments for outpatient THA are nearly double the payments in Germany, even though the German payment includes an average 7.2-day hospital stay vs no overnight hospital stay in the United States. US prices are also double those in Canada. The difference in payments between the United States and Canada appears to be driven by very large differences in facility fees ($5710 USD in Canada and $13 048 USD in the United States), with Canadian physician fees similar to those in the United States. Data from Germany did not separately identify physician fees and facility fees, as the DRG payments comprise both. This analysis focused on Medicare payments in the United States, which are readily accessible, but we note that commercial insurance payments for THA would likely be at least double those in Medicare.^44^

### Outcome reporting

Experts in Canada, England, and New Zealand indicated that Patient-Reported Outcome Measures (PROMs) are publicly reported for THA to encourage performance improvement. Experts in France noted that PROM reporting is expected to be publicly available by 2024, and in Germany a law requiring public reporting will go into effect in 2024.^45^

Canada, England, and France participated in an OECD pilot study to collect and report PROMs, including the Oxford Hip Score.^46^ The Oxford Hip Score assesses pain and functional status for patients undergoing THA across a 12-item inventory. Even within this pilot study, the measure calculations vary substantially across countries, with Ontario, Canada, reporting changes in Oxford Hip Scores across 12 months and England reporting changes across 6 months.^46^

In the United States, some US hospitals and orthopedic surgeons participate in the American Joint Replacement Registry, a voluntary organization that collects and reports a variety of data on joint replacements.^47^ Data submission is voluntary, and the recommended reporting for THA does not include the Oxford Hip Score as in other countries,^35^ although it may be possible to crosswalk the PROMs used in the United States to the Oxford Hip Score to allow for international comparison.^48^ The Centers for Medicare and Medicaid Services will also begin requiring mandatory collection of PROMs in the United States in 2027.^49^ In New Zealand, the New Zealand Orthopaedic Association runs a Joint Registry, which collects PROM data for hip replacement.^50^ In their 2023 report, the New Zealand Orthopaedic Association found that having an Oxford Hip Score below 27 was significantly associated with undergoing a hip revision within 2 years.^51^

## Discussion

Comparing THA processes of care in 7 high-income countries reveals positive and negative attributes of the US approach. The United States has high rates of outpatient THA, possibly reflecting an efficient approach to care, but also has notably higher prices than comparator countries. Germany, in contrast, has high volumes of THA and extremely long hospital stays, yet maintains THA payment rates that are well below those in the United States.

Because the majority of US hip replacements are covered by Medicare, this comparison is not as affected by the problem of uninsurance and underinsurance that is more predominant in United States among the privately insured population under age 65. However, we note that older-adult Medicare beneficiaries in the United States report being more likely to forgo care due to costs than older adults in other high-income countries.^52^

Despite concerns commonly raised in the United States that countries with universal health insurance and government regulation of prices and spending may ration care or limit the choices of patients and doctors, we found very similar processes for eligibility, surgical approach, and prosthesis selection across all 7 countries. In most cases, our experts indicated that the selection of surgical approach and prosthesis is driven by surgeons or a combination of surgeons and patients.

Similarly, experts reported wait times of 3–6 months in most countries, with the exception of shorter waits in Germany and longer waits in England and New Zealand. Reported average wait times were the shortest in Germany, with surgery available in as little as 2 weeks. However, wait times vary significantly within a country, and our experts reported that patients can lower their wait time by selecting an alternative surgical site. This suggests that the availability of surgeons and hospital beds may play a significant role in wait times. Overall, in all countries except for Germany, THA for noncomplicated patients results in a brief hospitalization followed by discharge home. The United States and Canada perform many surgeries with same-day discharge, while other countries still rely on an inpatient approach.

However, despite having an efficient THA process in place that minimizes hospital stays, the United States still appears to be an outlier in price. While costs and prices are not directly comparable across countries, they show a pattern of extremely high spending per case on THA in the United States compared with other high-income countries. This is consistent with longstanding literature that high US health care spending may be more a reflection of high prices than high utilization or inefficiency.^53,54^ Medicare payments for an outpatient THA in the United States are nearly double the payment for an inpatient THA in Germany, despite the payment in Germany including a long hospital stay.

While the United States is an outlier on cost per THA, Germany is an outlier on volume and intensity. In Germany, patients stay 7–9 days in the hospital after uncomplicated surgery and then receive inpatient rehabilitative care for several weeks. Germany also has among the highest rates of THA in the OECD (Table 1). This may reflect both excess hospital capacity^55^ and statutory entitlements to rehabilitative care after THA.^40^

The longer hospital and rehabilitative stays in Germany could allow for more comprehensive physical therapy, but they also risk exposing patients to hospital-acquired infections.^56^ There is some evidence that Germany has higher hospital-acquired infection rates than peer countries.^57^ Additionally, mobilization as soon as possible after surgery is associated with shorter LOS with no negative effects on outcomes.^58^ A quick discharge home allows for quicker rehabilitation and earlier resumption of normal activities. One paper found better outcomes in a US-based health care system with short hospital stays than in Germany, for example.^17^

Many countries are planning or testing alternative payment models for THA and public reporting approaches to improve quality or reduce costs. Canada, England, France, and New Zealand are all moving toward reporting PROMs, such as improvement in function post-surgery. US surgeons and hospitals can report these outcomes voluntarily, and they will be required in Medicare starting in 2027.^49^ The United States is also testing payment methodologies to reduce costs and potentially improve quality, but these approaches have had only small effects on reducing costs per THA case.^43^

The differences in THA rates across countries may reflect overuse in some countries. For example, in the United States, as many as one-third of joint replacements may be unnecessary, resulting in excess spending and exposing patients to surgical risk.^59^ The models being tested in Medicare generally do not address volume, only per-case costs. Other countries have developed payment system approaches to reducing unnecessary THA surgeries. For example, our experts noted that Canada has a cap on the number of THAs that hospitals can perform. Hospitals exceeding that cap receive reduced payments per THA. However, such an approach could potentially reduce access to care if the cap is set too low. Future research could explore variations in approaches to presurgical management of osteoarthritis across countries that may lead differences in THA rates, including rates of potentially unnecessary THA.

## Conclusion

Despite concerns raised in the United States about rationing of elective care in countries with universal health coverage, we found no evidence that physician decisions on referring patients for surgery, recommending a surgical approach, and selecting a prosthesis are significantly driven by government requirements across study countries. Furthermore, the United States has THA wait times in line with many of the other countries in our study. Overall, concerns about rationing of elective care appear to be misplaced. Total hip arthroplasty care in the United States proceeds in a very similar manner to care in the other countries in our study, except for Germany, albeit at a far higher price.

## Supplementary Material

qxae043_Supplementary_Data
