# Supplementary material for: Variation in processes of care for total hip arthroplasty across high-income countries
Source: Health Aff Sch. 2024 Apr 24;2(4):qxae043. doi: 10.1093/haschl/qxae043 (PMC11060656; doi:10.1093/haschl/qxae043)
Supplement: qxae043_Supplementary_Data [file qxae043_supplementary_data.zip › Appendix 1.docx]

**Appendix 1**

**Total Hip Arthroplasty Questionnaire**

This interview guide is separated into five sections: eligibility and scheduling; surgery; post-acute care and rehabilitation; outcomes tracking; and payment. For the first three sections, unless otherwise noted, please base your responses on **typical care for a 68-year-old male with osteoarthritis** and no complicating chronic conditions. Please assume this patient is insured through the dominant system in your country (Medicare in Ontario, Canada; CNAM in France; Public Health Insurance (GKV) in Germany; public healthcare in New Zealand; Folketrygd in Norway; National Health Service in the UK; and Medicare in the US).

We are interested in your view of what is typical in your country or region. If there are important, systematic variations in typical care in your country, such as by region or insurance type, please briefly describe where appropriate.

**Section 1: Eligibility and Scheduling**

1. Please indicate the dominant insurance/coverage system in your country or region for total hip arthroplasty (THA) for a typical 68-year-old male and indicate the share of similar patients covered under that system, if known.
2. What share of THA surgeries are delivered privately (i.e. through private insurance and/or private hospital systems), if any?
3. Are the orthopedic surgeons who perform total hip arthroplasty on patients with the dominant insurance type in your county typically hospital employees or in private practice? What about orthopedic surgeons who perform THA on privately insured patients?

1. What is the typical referral process a 68-year-old male with osteoarthritis and no complicating chronic conditions who is a candidate for THA?
   1. The patient’s primary care physician refers them to an orthopedic surgeon or practice
   2. The patient self-refers to an orthopedic surgeon, surgical practice, or hospital
   3. The patient’s insurance company refers them to an orthopedic surgeon, surgical practice, or hospital
   4. Some other approach (please describe)
2. What are the eligibility criteria, if any, for insurance coverage of elective total hip arthroplasty in your country (e.g. age, degree of arthritis) and how rigorously are they enforced? Please provide a link to documentation of eligibility criteria, if available.
3. Are there any formal shared decision-making processes in place to help patients participate in decision-making about the appropriate treatment for their osteoarthritis? Formal shared decision-making processes could include patient questionnaires, decision aides, or other tools to help patients understand their options and voice their preferences.
4. If the patient and an orthopedic surgeon agree on the need for total hip arthroplasty, how often are patients denied coverage for the procedure?
   1. Never
   2. Rarely
   3. Sometimes
   4. Often
   5. Something else
5. If patients fail to meet the eligibility criteria for insurance coverage, if applicable, can they elect to receive surgery through private insurance, a private hospital, or self-funded means?
6. Once a patient is determined eligible, how does scheduling of the surgery proceed? Are there typically waiting lists for surgery that extend beyond one month? If there are not typically waiting lists that extend beyond one month, please skip to question 13.
7. How long is the current wait time from referral to surgery for a 68-year-old male with osteoarthritis and no complicating conditions in your country or region? Are wait times fairly comparable within a geographic area, or do they vary by hospital, surgeon, or insurance type?

1. Can patients reduce their wait time through any of the following:
   1. Seeking an alternative surgical site, such as a different hospital or surgical center covered within the dominant insurance plan
   2. Selecting a private hospital (in countries with publicly-funded hospital systems)
   3. Purchasing supplemental insurance
   4. Paying out of pocket
   5. Something else (please describe)
2. Are wait times tracked by national, local, or regional governments? Please provide a link to any publicly available wait time information, if possible.
3. Are there any limitations on which hospitals and/or surgeons are allowed to perform total hip arthroplasty?

1. Are there any publicly available websites or databases, whether government-funded or private, that provide patients with cost, quality, outcomes, or waiting time information? If so, please provide a link to the websites, if possible.

**Section 2: Surgery**

1. Who typically decides the type of THA procedure (e.g. cemented, hybrid, or uncemented hip prosthesis) and surgical approach (anterior vs posterior) to be used in your country or region? If multiple answers apply, please describe the decision-making process. Please also note any patient involvement in the decision-making process
   1. The orthopedic surgeon determines the appropriate procedure type and approach
   2. National or regional care guidelines specify the appropriate procedure type and approach
   3. Hospital guidelines specify the appropriate procedure type and approach
   4. Insurer or payer guidelines specify the appropriate procedure type and approach
   5. Other (please describe)

1. Who typically selects the type of prosthesis/bearing surface that will be implanted (e.g. metal-on-polyethylene, ceramic-on-ceramic, etc)? If multiple options apply, please describe the decision-making process.
   1. The surgeon selects the bearing surface/prosthesis
   2. The surgeon recommends an appropriate bearing surface/prosthesis to the patient and the decision is made jointly
   3. National or regional care guidelines specify an appropriate bearing surface/prosthesis
   4. Hospital guidelines specify an appropriate bearing surface/prosthesis
   5. Insurer/payer guidelines specify an appropriate bearing surface/prosthesis
   6. Other (please describe)
2. Do patient out-of-pocket costs differ based on the procedure type, surgical approach, or bearing surface?
3. How long would a 68-year-old with uncomplicated THA typically spend in the hospital immediately following surgery? If hospital stays vary substantially by region or hospital, please describe the typical range. Please provide links to data or research on hospital lengths of stay if possible.
   1. Discharged same day without an overnight stay
   2. 1 day
   3. 2-3 days
   4. 4-5 days
   5. 6-7 days
   6. More than 7 days
4. Do any of the following commonly lead to changes in the planned location or timing of discharge? If yes, please describe.
   1. Geography/region
   2. Ability to return for follow up care
   3. Who the patient is living with, or if the patient is living alone
   4. Patient receptivity and preferences
   5. Housing situation, including presence of stairs
   6. Resources in the community (e.g. availability of physical therapy, availability of follow-up care)
   7. Insurance type (public vs private)

**Section 3: Post-Acute Care and Rehabilitation**

Unless otherwise noted, in this section please base your responses on a typical 68-year-old male with osteoarthritis and no complicating conditions who had a successful THA.

1. Approximately what share of patients are discharged to the following locations after an uncomplicated THA? If there are important regional variations in discharge location, please note them. Please also provide links to publicly-available data or research where available.
   1. Home
   2. A rehabilitation facility or other post-acute care facility (please describe)
   3. Some other medical facility (please describe)
   4. Somewhere else (please describe)
2. What types of care would a 68-year-old male with an uncomplicated THA typically receive in the month following surgery? Please select all that apply and briefly describe.
   1. Inpatient rehabilitative care
   2. Outpatient physical therapy
   3. Home-based physical therapy
   4. Outpatient occupational therapy
   5. Home-based occupational therapy
   6. Follow-up visits with the operating surgeon or another affiliated surgeon
   7. Follow-up visits with primary care doctors or non-physician clinicians (e.g. nurse, nurse practitioner, physician’s assistant)
   8. Follow-up visits with other providers (please specify)
3. Who typically coordinates rehabilitative care for a 68-year-old male with uncomplicated THA?
   1. The hospital
   2. The operating surgeon
   3. The patient’s primary care doctor
   4. A care coordinator
   5. The patient
   6. Someone else (please describe)
4. If inpatient rehabilitation (e.g. in a rehab facility or other post-acute care facility) is provided after THA surgery, how long would a THA patient typically spend in inpatient rehabilitation? Please provide a link to any public reporting or research on inpatient rehabilitation length of stay.
5. If outpatient or in-home physical or occupational therapy is provided after THA surgery, how long after discharge does therapy usually begin?
   1. Within 1-2 days of discharge
   2. Within 3-7 days of discharge
   3. Within 2-3 weeks of discharge
   4. More than 3 weeks after discharge
6. How many physical therapy sessions would an uncomplicated THA patient typically receive in the 3 months following surgery?
7. Does the rehabilitative process and location differ substantially for patients with uncomplicated THA in rural areas? If so, how?
8. What role do patient preferences play in the clinical setting and duration of rehabilitative care or of the specific facility referred to?
9. Are any decision aides, public data, or other supports available to inform patient choices of rehabilitation location (e.g. comparisons of rehab facilities, formal shared decision-making processes for selecting home versus inpatient rehab). If so, please provide a link to any relevant documents and websites.

**Section 4: Outcomes Tracking**

1. What public reports, if any, are available at the hospital, regional, or national level to monitor and assess THA use and both clinical and patient-reported outcomes? How are these reports used, if at all? Please provide a link to any public reporting on THAs, if available.
2. Are outcomes tracked or reported by patient demographics, such as race and ethnicity, age, gender, region, or across different public and private insurers? If so, please provide a link to any public reporting websites or document.
3. Is performance on outcome measures used for any of the following? If so, how?
   1. Payment
   2. Public reporting
   3. Other provider or hospital performance incentives

**Section 5: Payment**

1. Is THA covered under global hospital payments, or do hospitals and/or physicians receive separate payments for each THA they perform? For the purposes of this question, episode-based payments are considered separate payments for services provided over an extended period of time. DRG-based payments subject to global hospital caps are also considered separate payments.
   *Note: If THAs are covered under global payments, please skip the remainder of the questionnaire.*

1. What is the typical THA payment rate for both professionals and for hospitals, if not combined into a single payment? If unknown, please indicate any potential sources of payment data that you may be aware of.
2. Please describe any payment approaches used or being tested for THA in your country to reduce costs or increase value (e.g. episode-based payments that cover multiple services performed in a specified time period, performance-based payments that include bonuses or penalties for care quality and/or outcomes). Please note any data our research describing the results of these alternative payment methods.
