# Supplementary material for: Variation in processes of care for total hip arthroplasty across high-income countries
Source: Health Aff Sch. 2024 Apr 24;2(4):qxae043. doi: 10.1093/haschl/qxae043 (PMC11060656; doi:10.1093/haschl/qxae043)
Supplement: qxae043_Supplementary_Data [file qxae043_supplementary_data.zip › Appendix 2.docx]

**Appendix 2**

**Number and Type of Expert Responses Received from Each Study Country**

| **Country** | **Number of Expert Questionnaire Responses Received** | **Areas of Expertise for Respondents** | **Regions/Cities for Respondents** |
| --- | --- | --- | --- |
| Canada | 4 | Surgery/medicine, Physiotherapy, healthcare administration | Ontario (2), northern Ontario, Nova Scotia |
| France | 2 | Surgery/medicine, policy, research | Paris |
| Germany | 2 | Post-acute care administration, hospital administration, surgery/medicine | Hamburg |
| New Zealand | 3 | Surgery/medicine | Auckland, Bay of Plenty, Southland |
| Norway | 2 | Surgery/medicine, research | Lillehammer, Oslo |
| UK | 3 | Surgery/medicine, research | Birmingham, Oxford (2) |
| US | 3 | Surgery/medicine, research | Northeast |

Note: Expert responses were supplemented with secondary data sources and literature. Wherever possible, national data for each country were used.
